# Chair Size Design Based on User Height

**DOI:** 10.3390/biomimetics8010057

**Published:** 2023-01-31

**Authors:** Maciej Sydor, Miloš Hitka

**Affiliations:** 1 Department of Woodworking and Fundamentals of Machine Design, Faculty of Forestry and Wood Technology, Poznań University of Life Sciences, Wojska Polskiego 28, 60-637 Poznań, Poland; 2 Department of Economics, Management and Business, Faculty of Wood Sciences and Technology, Technical University in Zvolen, T. G. Masaryka 24, 960 01 Zvolen, Slovakia

**Keywords:** anthropometry, furniture design, chairs, seat width, seat depth, seat height, lumbar support

## Abstract

General principles derived from anatomical studies of human body sizes should be applied to chair designs. Chairs can be designed for a specific user or a particular group of users. Universal chairs for public spaces should be comfortable for the largest possible group of users and should not be adjustable, such as office chairs. However, the fundamental problem is that the anthropometric data available in the literature either come from many years ago and are out of date or do not provide a complete set of all the dimensional parameters of a sitting human body position. This article proposes a way to design chair dimensions solely based on the height range of the intended chair users. For this purpose, based on literature data, the main structural dimensions of the chair were assigned to the appropriate anthropometric body measurements. Furthermore, calculated average body proportions for the adult population overcome the incompleteness, outdated and burdensome access to anthropometric data and link the main chair design dimensions to one easily accessible anthropometric parameter: human height. This is achieved by seven equations describing the dimensional relations between the chair’s essential design dimensions and human height or even a height range. The result of the study is a method of determining the optimal functional dimensions of a chair for a chosen range of sizes of its future users based only on users’ height range. Limitations of the presented method: the calculated body proportions are correct only for people with a standard body proportion characteristic of adults, i.e., they exclude children and adolescents up to 20 years of age, seniors, and people with a body mass index exceeding 30.

## 1. Introduction

The characteristic feature of biomimetics is that it uses biological information to obtain a proper technical implementation. The usage of biomimetics in product development starts by linking a biological system to a specific technical question [1] and goes through conceptualization, reading out of the biological properties, modeling of these properties, modification of the model, and its application. Human body size is a vital biomimetic property used in furniture design. The influence of biomimetics on a furniture product may determine the essence of the product or may have a small impact on one feature of the product.

Spending too much time sitting is not recommended due to the risk of health problems. Even people who exercise regularly should not stay in a sitting position for too long. A person can get enough physical activity and still lead a sedentary lifestyle if they spend too much time sitting at school, at work, traveling, or in their free time. This increases the risk of many health problems, including obesity, cardiovascular disease, and spinal disease. Sitting too much in an uncomfortable position also affects human mental abilities, such as focusing of attention or acting effectively. These are solid arguments for designing all chairs following the dimensions of the person who sits on them.

Chairs are furniture intended for direct contact with people, designed as general-purpose furniture or for specific groups of users. Chairs are, therefore, a class of furniture, the sizes of which should be well suited to the sizes of the human body. In some cases, such as a wheelchair design, the seat and backrest are fitted to a single user. Then, other humans with different body sizes will not be able to use it comfortably [2]. In the case of office chairs, adjusting the chair’s functional dimensions fits the group of users. However, adjusting the chairs is not acceptable for universal chairs intended for many people in public spaces. In this case, the chair must be efficiently “averaged” in size. This “averaging” is based on its fit within the size range of its intended users. The choice of the dimensional range of users is the designer’s decision; it depends on the chair’s use, while the dimensional parameters of users from the selected dimensional range can be taken from anthropometric data.

The anthropometric data used in furniture design are statistically processed information about the human body’s sizes for engineering applications. A commonly referenced database used in engineering design is from military data collected between the 1970s and 1980s, known as the Natick studies or ANSUR database. In 2000, the Civilian American and European Surface Anthropometry Resource (CAESAR) was compiled by the Society of Automotive Engineers (SAE). CAESAR contains anthropometric data and 3D body scans of over 4000 individuals from North America and Europe. The Business and Institutional Furniture Manufacturers Association (BIFMA) and many ergonomics textbooks reference Natick (military) studies for design purposes, but some groups use CAESAR data or both the Natick and CAESAR datasets in design. More emphasis is being placed on the CAESAR database because it represents today’s office population more than the Natick databases. BIFMA and others still reference Natick’s measurements. Three points are vital when using anthropometric measurements in design: (1) How recently has the data been updated? (2) What was the type of population measured? (3) Are the anthropometric sizes complete from the point of view of the chair designer? Or can they be easily “translated” into the main design of the chairs? First, some data may have been collected more than 25 years ago, and measurements such as body height or body mass may have changed. Secondly, the referenced anthropometric database may not represent the planned users. For example, ANSUR data may not be applicable to designing chairs for seniors [3]. Third, the relevant information is missing from the database. Examples of missing parameters are buttock-popliteal length and lumbar height. The first parameter is essential when designing the depth of the seat, and the second is when designing the chair backrest.

The following resources provide information on anthropometry: internet databases (BIFMA, bifma.org; CAESAR, store.sae.org/Caesar; Size USA, sizeusa.com); anthropometric atlases of human measurements, e.g., [4,5,6] or books (Handbook of Human Factors and Ergonomics, 2012 [7], Human Factors Design Handbook, Tillman, Tillman, 2016 [8]); and digital human models (DHM) as standalone software or built into CAD systems. To include human anthropometric dimensions in a designed product, digital human models (DHM) are used as modules in well-known CAD programs. There are many DHMs; the most commonly used are JACK (ugs.com) and RAMSIS (human-solutions.com) [9]. For example, the SiemensNX CAD software uses JACK, Catia uses RAMSIS and HUMAN BUILDER, widely used in the interior design of cars [10,11]. Other DHMs used in design include DELMIA Safework (delmia.com), ErgoForms (ergoforms.com), LifeMOD Biomechanics Modeler (lifemodeler.com), and ManneQuinPRO (nexgenergo.com). DHMs allow the design of a product tailored dimensionally to a person of selected body dimensions or a larger group of people within the desired size range, analogous to anthropometric atlases [12]. Another source of information about human body proportions is an artistic canon of body proportions used in visual arts. It is commonly accepted that the standard height of the human body is 7.5 times the height of the head [13]. Visual arts textbooks also provide information about the proportions of the length of individual parts of the human body [14].

Both anthropometric atlases and DHMs potentially enable the design of products well-tailored to anthropometric data, but all of these data sources may be incomplete for chair designers. The secular trend in human height is known and well described in the literature; for example, studies on the Slovak population indicate that average height increases by 0.3–2.0 cm per decade [15]. Furthermore, using anthropometric data effectively requires ergonomic knowledge to correctly translate human dimensions into a chair design feature, for example, the length of the lower legs into the optimal height of the chair. In addition, in some anthropometric databases, some dimensions of a person in a sitting position are missing.

This paper, therefore, addresses two crucial issues related to obtaining an ergonomic chair for a chosen human subpopulation:

1. How properly to link the functional dimensions of a chair with the human body’s sizes;
2. How to deal with the lack of some data in the anthropometric database used.

The starting point for the analysis presented in the article is the statement that it is possible to link all “ergonomic” dimensions of a chair solely to human height. Moreover, as a result, it enables the design size of the chair for public spaces to be based only on user height.

## 2. Chair Dimensioning

**Figure 1:**
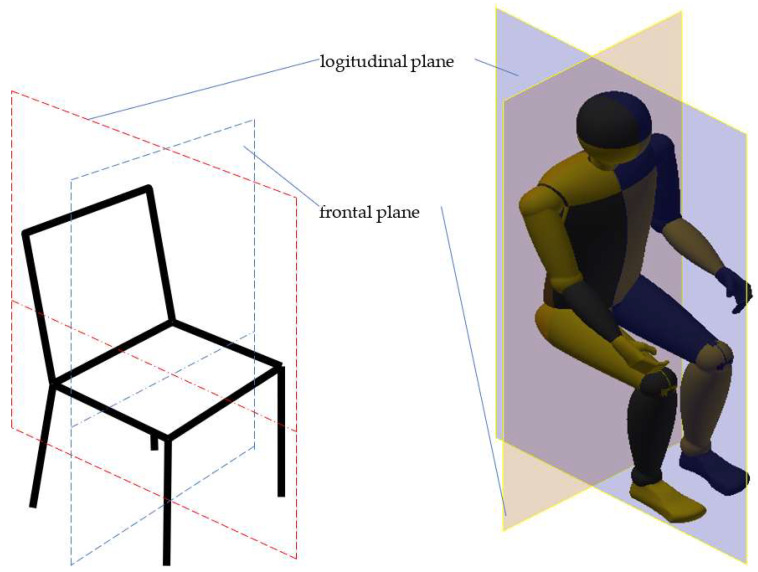
Representation of the longitudinal (median) and frontal cardinal planes in chair design (inspired by EN 1729-1:2015) and humans in the sitting position.

There are three cardinal planes of the human body [16]. Two of them are particularly helpful in right chair dimensioning: the longitudinal (sagittal) plane, dividing the left and right sides of the body, and the frontal (coronal) plane dividing the body into a front (anterior) section and back (posterior) section [17]. Figure 1 presents these two cardinal planes.

**Figure 2:**
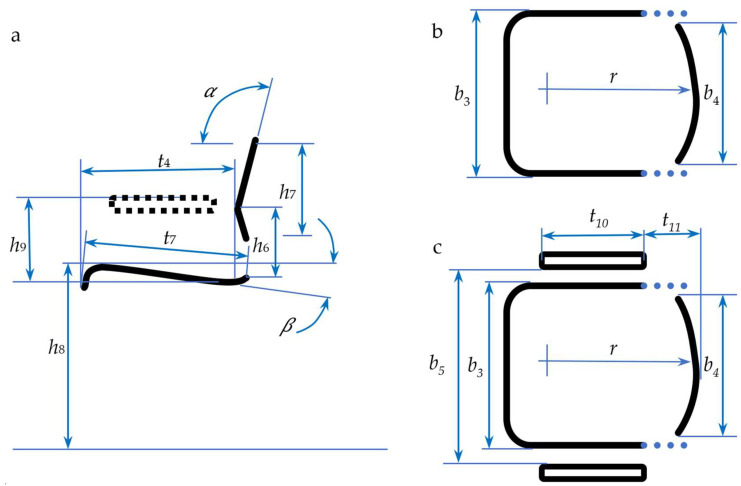
The functional dimensions of a chair (𝛼—backrest angle, 𝛽—seat angle, *h*_8_—seat height, *b*_3_—seat width, *b*_4_—backrest breath, *b*_5_—armrest spacing, *h*_6_—lumbar support height, *h*_7_—backrest height, *h*_9_—armrest height, *r*—backrest radius, *t*_4_—seat depth normal, *t*_7_—seat depth, *t*_10_—armrest length, and *t*_11_—armrest to seat length): (**a**) side view, dimensions in a longitudinal plane, (**b**) top view on a chair without armrests, (**c**) top view on a chair with armrests.

Figure 2 shows the functional dimensions of a chair according to the two cardinal human body planes.

**Figure 3:**
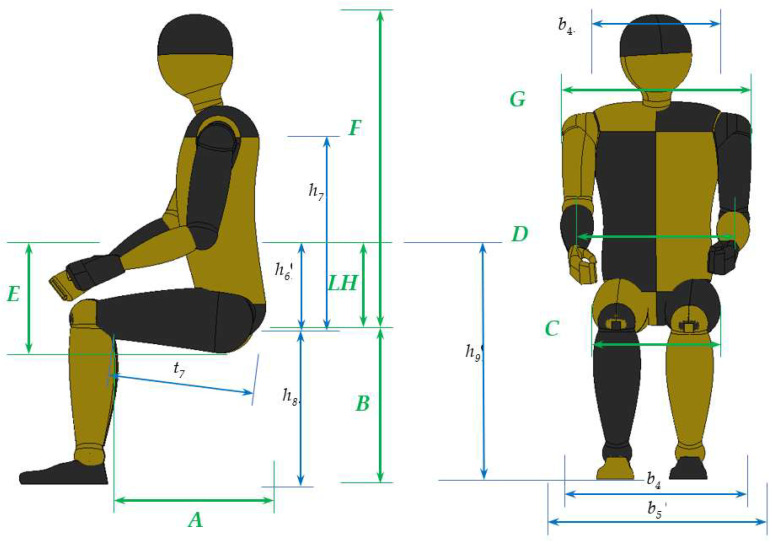
The functional dimensions of a chair and the anthropometric measurements.

The functional dimensions of a chair should be correlated with the anthropometric body sizes of the planned group of chair users. Figure 3 shows these anthropometric measurements and the main design dimensions of the chair.

## 3. Methodology: A Proposal for Linking the Functional Dimensions of a Chair to the Anthropometric Measurements

The main difficulty in proper chair design is the correct connection between the human body’s sizes and the functional dimensions of a chair. Table 1 shows a proposal for such a connection.

The challenge in designing chairs for the general public is the anthropometric diversity of the human adult population. There are sex differences in stature and related varieties in body dimensions and bodily proportions. These differences are almost entirely biological in their origin, although there may be a slight overlay of differences associated with physical training and lifestyle. Longitudinal anthropometric studies show that humans begin to shrink in stature at around 40 years of age; women shrink more than men [15]. Involution changes in the intervertebral discs of the spine mainly cause this shrinkage. The shrinkage is observable as the characteristic round back of the elderly. Another source of diversity is the “secular trend”. The “secular trend” refers to an alteration in the measurable characteristics of a population occurring over time. Biosocial changes increase the growth rate of children, and an earlier adolescent growth spurt in both boys and girls increases adult stature, with a possible decrease in the age at which adult stature is reached. The percentage of obese people is increasing, which requires changes in chairs’ dimensions and load capacity for general use. The magnitude of the “secular” changes in different countries is non-uniform. The secular changes can vary greatly from country to country, depending on various factors, such as cultural, economic, political, and historical influences.

**Figure 4:**
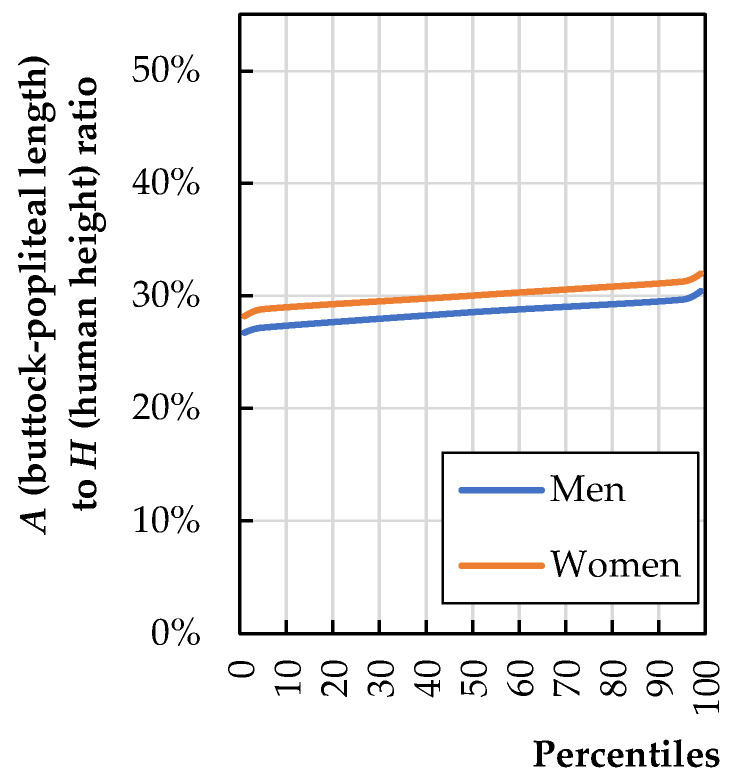
Ratio of buttock-popliteal length (*A*) to total human stature (*H*) (calculations based on literature data [20]).

The maximum seat depth of a chair (*t*_7_) is limited by the length of the thighs of the shortest persons using the seat (*A*—buttock-popliteal length). According to Ravindra et al. [18], the optimal seat depth should be 10 cm shorter than the median buttock-popliteal length BPL of the users. A seat too deep makes it difficult to use the backrest, which causes discomfort when sitting [19]. Too large a seat depth affects blood circulation to the legs; therefore, compression of the tissues causes discomfort. A too-shallow seat depth results in lack of support for the lower thighs and causes the sensation of falling off the front of the chair. Considering all of this, it is reasonable to design the *t*_7_ dimension of the chair based on the height of the smallest expected chair user. Figure 4 shows the ratio of dimension A to human height. The dimension ratio of A to H was calculated based on anthropometric data for the U.S. population published by Stoudt et al. [20].

Figure 4 shows that the anthropometric dimension “*A*” of a 5th-percentile woman is 29% of her height ($H_{5\mathrm{th}\;\mathrm{women}}$). This can be written as Equation (1):

(1) $$t_{7}=0.29\cdot H_{5\mathrm{th}\;\mathrm{woman}}$$

More recent anthropometric data for the U.S. population, published by Fryar et al. [6], covering data collected in the years 2007–2010, show that the height of a 5th-percentile woman is 150.4 cm, so the dimension *A* calculated with Equation (1) and, at the same time, the dimension *t*_7_ of the chair, is 43.6 cm.

The height of a chair seat (*h*_8_) should be no more than the popliteal height (*B*) of the shortest chair user, because, in sitting, the trunk’s weight should be borne mainly by the ischial tuberosities. The thighs are anatomically and physiologically unsuited for supporting the body weight. However, there is some conflict between the need to design a seat height low enough to accommodate shorter people and the need to avoid excessive hip flexion and the convexity of the lumbar spine that accompanies it in the taller person sitting on a seat [21]. According to the anthropometric data provided by Stoudt et al. [19], the popliteal height of men in the 95th-percentile is 490 mm, while this parameter for a woman in the 5th-percentile is only 33.6 cm. A reasonable compromise in this situation seems to be to design the *h*_8_ dimension according to the height of a 50th-percentile woman and add 2 cm as the standard thickness of the sole of the shoe.

**Figure 5:**
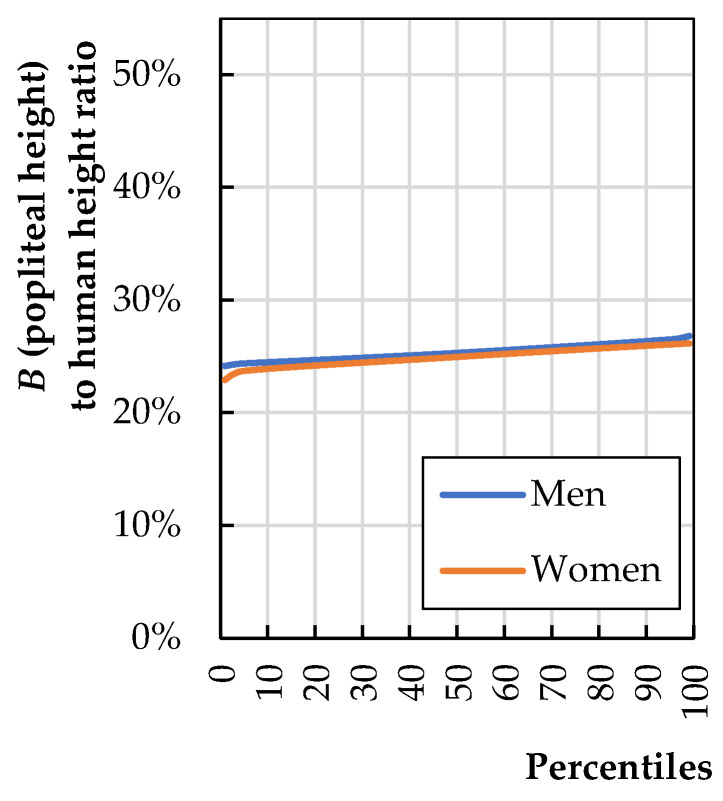
The ratio of buttock-popliteal length (*B*) to total human stature (*H*) (calculations based on literature data [20]).

Figure 5 shows the ratio of popliteal height (*B*) to human height (*H*). The calculations on the figure were made based on anthropometric data published by Stoudt et al. [20].

Figure 5 shows that the anthropometric dimension “B” of a 50th-percentile human is 25% of their height, which can be written as Equation (2):

(2) $$h_{8}=0.25\cdot H_{\mathrm{average}}+2\;\mathrm{cm}$$

Using Equation (2) and more recent anthropometric data from Fryar et al. [6], it can be calculated as *h*_8_, based on a modern 168.6 cm-tall 50th-percentile human, as 44.2 cm.

Seat width (*b*_3_) is designed including trochanteric width (*C*), plus an excess on both sides (for example, 6 + 6 cm [22]). A standard for school seating furniture, EN 1729:2015 [17], proposes a seat width for the highest students with a height of 207 cm, *b*_3_ = 40 cm. Malik et al. [19] postulate a seat width of 40 cm for the working chair but recommend at least 45 cm for a general-use chair. The seat width for bariatric users includes a widened range of 55.9–73.0 cm. ANSI/BIFMA X5.41-2021, [23] for the 99th centile, postulated a minimum seat width of 22 inches (55.9 cm) for the 400 lb user and 26 inches (66.0 cm) for the 600 lb user. Hitka et al. [22] recommend seat widths of 67 cm for 95th-centile bariatric users and 73 cm for 99th-centile bariatric users. However, when designing a general-use chair, it seems rational to set the seat width as the hip width of the largest anticipated user of the chair, increasing the *b*_3_ with small excesses on both sides.

**Figure 6:**
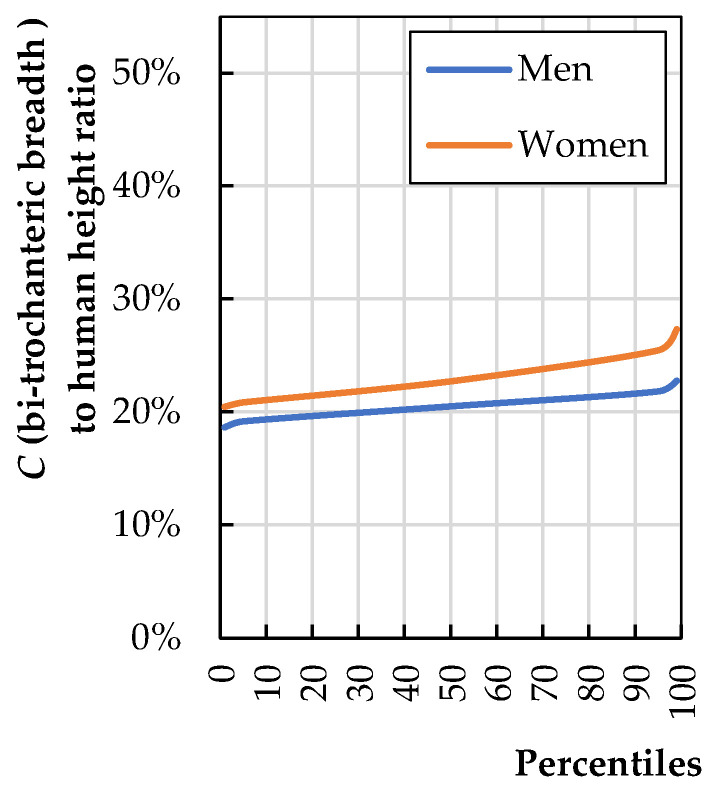
Ratio of bi-trochanteric breadth (*C*) to total human stature (*H*) (calculations based on literature data [20]).

Figure 6 shows the ratio of the anthropometric dimension C to height (H). The largest bitrochanteric breadth (or biiliac breadth) among the 5–95th-percentile user range is from the 95th-percentile woman, and such a woman has a hip width of 25% of her height (the calculations were made based on data provided by Stoudt et al. [20]).

Assuming two-centimeter spacings on both sides, Equation (3) can be written:

(3) $$b_{3}=0.25\cdot H_{95\mathrm{th}\;\mathrm{woman}}+2\cdot 2\;\mathrm{cm}$$

Using Equation (3) and based on a contemporary 173.1 cm-tall 95th-percentile woman [6], *b*_3_ = 47.3 cm.

**Figure 7:**
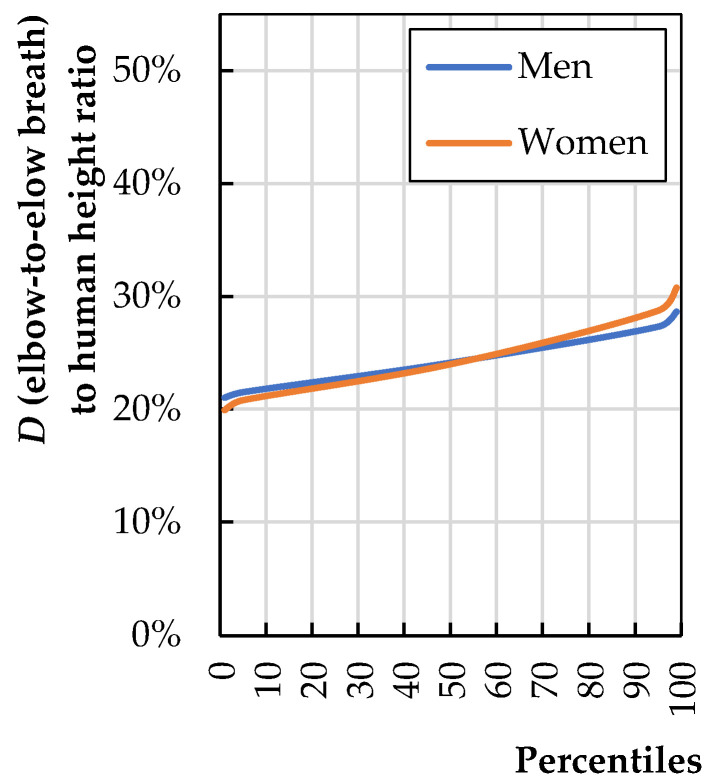
Elbow-to-elbow breath (*D*) to total human stature (*H*) (calculations based on literature data [20]).

If the chair has armrests, their spacing (*b*_5_) should be based on elbow-to-elbow breath (*D*). Armrest spacing should also be correlated with trochanteric width (C) and equal to or broader than the seat width (*b*_3_). Figure 7 shows the ratio of dimension *D* to human height (*H*). The calculations were made based on data published by Stoudt et al. [20].

In this case, the largest user is a 95th-percentile male. The ratio of the anthropometric dimension *D* of such a man to his height is 27%. Since the spacing of the armrests should not restrict the movements of the seated person, it is reasonable to use increased spacing on both sides of up to 3 cm.

(4) $$b_{5}=0.27\cdot H_{95\mathrm{th}\;\mathrm{man}}+2\cdot 3\;\mathrm{cm}$$

Using Equation (4) and anthropometric data from Fryar et al. [6], one can calculate *b*_3_ based on a modern 187.7 cm-tall 95th-percentile man as 66.7 cm.

**Figure 8:**
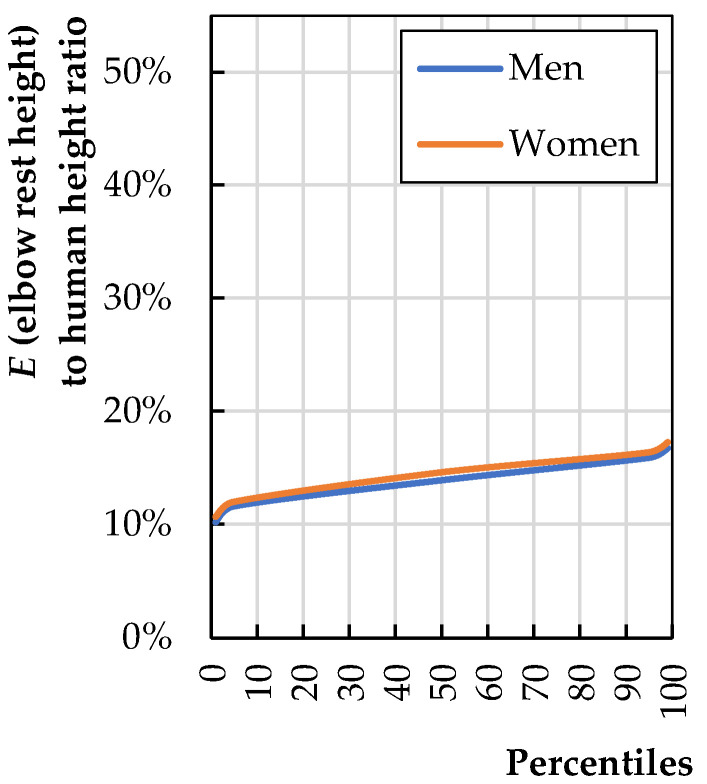
Elbow rest height (*E*) to total human stature (*H*) (calculations based on literature data [20]).

Armrest height (*h*_9_) should equal elbow rest height (*E*). Figure 8 provides the ratio of dimension *E* to human height (*H*); the calculations were made based on data provided by Stoudt et al. [20].

In this case, using the mean value for the entire population seems reasonable. Dimension *E* is 14% of the height.

(5) $$h_{9}=0.14\cdot H_{\mathrm{average}}$$

Using Equation (5) and anthropometric data from Fryar et al. [6], one can calculate *b*_3_ based on a modern 168.6 cm-tall 50th-percentile human as 23.5 cm.

**Figure 9:**
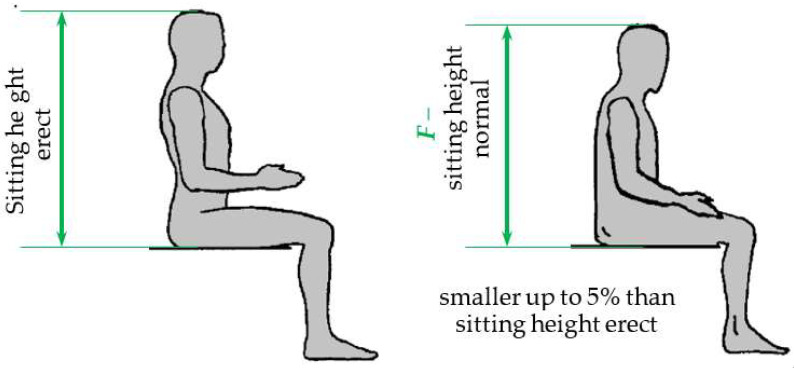

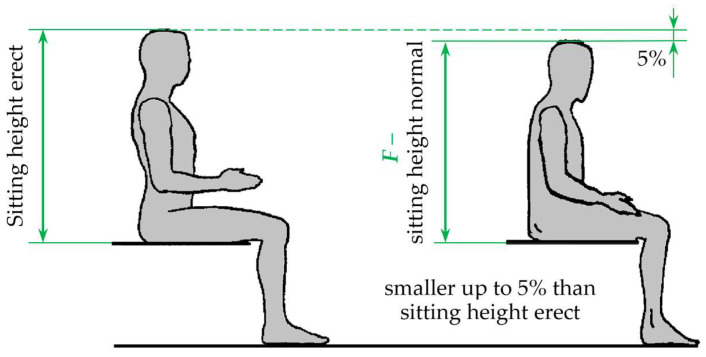
Sitting height erect versus sitting height normal.

The height of the backrest (*h*_7_) of the chair is correlated with the anthropometric dimension *F* (sitting height, i.e., the sum of the lengths of the trunk, neck, and head). This dimension has two variants, sitting height erect and sitting height normal. The first variant is characteristic of sitting in an office chair with lumbar support. The second, when a person sits in a natural, relaxed position, is more appropriate in the design of chairs. The difference between the two sitting heights is shown in Figure 9.

Based on anthropometric data, for example, for the body dimensions of adults in the USA [20], it can be calculated that sitting height normal is smaller by 2–5% than sitting height erect.

**Figure 10:**
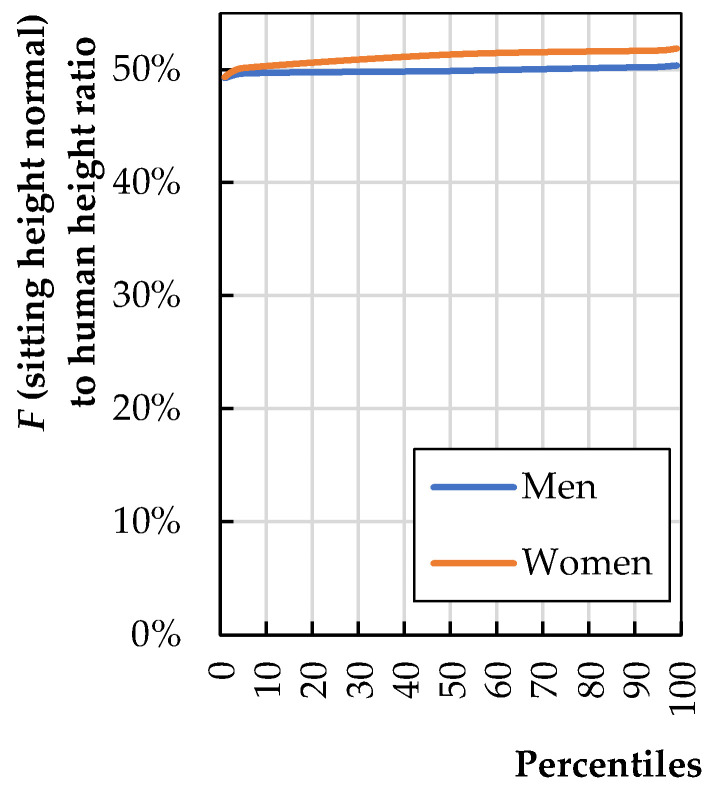
Sitting height normal (*F*) to total human stature (*H*) (calculations based on literature data [20]).

Figure 10 provides the ratio of dimension *F* to human height (*H*); the calculations are based on data provided by Stoudt et al. [20].

Figure 10 shows that the sitting height normal is 50% of the height. The backrest height depends on the chair’s expected sitting position. It seems that a universal chair for general use should be 75% of the dimension *F* for an average person.

(6) $$h_{7}=0.50\cdot 0.75\cdot H_{\mathrm{average}}$$

Using Equation (6) and anthropometric data from Fryar et al. [6], one can calculate *h*_7_ based on a modern 168.6 cm-tall 50th-percentile human as 63.2 cm. The backrest can be higher. The height should be increased if the backrest angle 𝛼 is greater than 105°.

**Figure 11:**
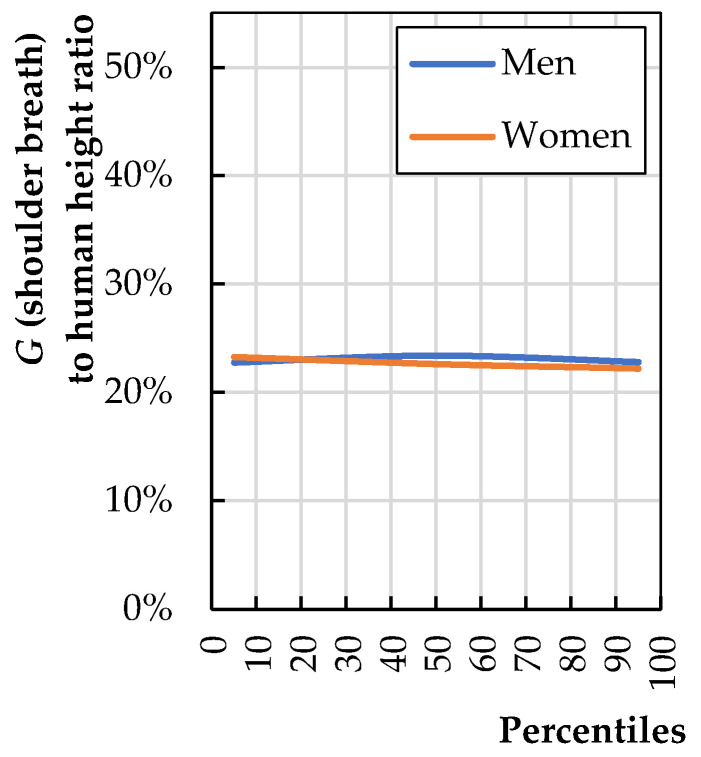
Shoulder breath (*G*) to total human stature (*H*) (calculations based on literature data [20]).

The backrest breath (*b*_4_) should include the biggest user. Figure 11 shows the ratio of dimension *G* to human height (*H*); the calculations are based on data provided by Fryar et al. [6].

(7) $$b_{4}=0.23\cdot H_{95\mathrm{th}\;\mathrm{man}}$$

Using Equation (7) and anthropometric data from Fryar et al. [6], one can calculate *b*_3_ based on a modern 187.7 cm-tall 95th-percentile man as 43.2 cm.

A plane seat is preferable to one shaped or molded to fit the backside. In sitting, the possibility of changes in posture is essential. A good chair should permit this. A backward slope of the seat (𝛽) may be suitable. However, a backward slope of up to 5° increases the comfort of sitting, but it can make it difficult rising from a seat, especially for seniors. Trunk muscle activity in sitting is diminished by using a backrest, which should not restrict the movement of the spinal column or arms. A lumbar support height (*h*_6_) within the limits of the second to fifth lumbar vertebrae allows adequate free movement. With height in the range of 146–207 cm, the standard EN 1729-1 recommends *h*_6_ in the 20–22 cm range. A saddle-shaped backrest (*r*) is recommended because it provides adequate side support for a trunk. The standard EN 1729-1 recommends a minimum value for this parameter of 30 cm [17]. An example value is *r* = 120 cm.

In addition to dimensions, other elements affect the comfort of the chair. The mechanics of ascending from a seat require that horizontal struts or other obstructions be placed between the front legs of a chair. The table and chair form a single anthropometric unit. Table height is closely correlated with elbow height. The elbows should be at about the level of the table’s working plane. The space between the under-surface of the table and the chair seat should be slightly greater than the thigh thickness.

## 4. Discussion

### 4.1. A Novel Finding of This Study

Some studies of chair ergonomics provide the oldest scientific arguments for ergonomic chair backrest design [24]. An EMG study of muscle tensions and spinal intra-disc pressure demonstrates that disc pressure in sitting increases by about 50% compared to standing due flattening of the lumbar curve [25]. To reduce disc pressure, a chair designer restores the lumbar curve by lumbar support or by increasing the backrest angle to 120° [26]. There are additional design criteria: (1) the chair front should not cut off the blood circulation in the legs, and (2) the chair must dimensionally fit the user’s size.

The matching of the dimensions of chairs is well-suited for school furniture. However, some studies report that the equations used to match school chairs and anthropometric dimensions are problematic because they are based on contradictory criteria [27,28,29].

The data for designing the dimensions of the chairs are recorded in the relevant standards, e.g., in the quoted standard for school furniture EN 1729-1:2015 [17]. However, they are static. They cannot simply be applied to any subpopulation of people. In this article, we propose a different approach. The “active” dimensioning of the chair during design based on easily obtained anthropometric dimensions benefits the presented approach. This methodology can also consider the different proportions of the human body (in the example, specific body proportions were used, calculated based on a selected database).

### 4.2. Study Limitations

The calculations of body proportions used in Equations (1)–(7) are based on a selected database for adults, without division by age, gender, ethnicity, or other differentiating factors. The database selection limits the calculation results: the results presented in this article are correct for people with normal body proportions characteristic of adults, i.e., they do not include:

- Children and adolescents under 20, who have specific ages’ body proportions;
- Seniors, because there is a general tendency of human height to decrease with age. This body shortening changes the proportions of the body because the shortened torso is mainly responsible for the reduction in height [30]. Women shrink more than men with age [15];
- There are significant differences in body proportions between geographically diverse human populations [31,32];
- The results also do not apply to people whose body proportions significantly differ from the average due to other causes, e.g., due to genetic conditions, physical activity, specific lifestyle, or disability.

These limitations can be eliminated by using a more specialized anthropometric database. It is then possible to modify the proportionality coefficients given in Equations (1)–(7) and, as a result, obtain formulas for designing chairs for people with different body proportions.

It is worth mentioning that there are significant differences in body proportions between men and women [33]. The proposed method averages out the differences and makes it possible to design “averaged” chairs for public spaces. They may be uncomfortable for people of extreme height, e.g., very small or very tall.

## 5. Summary and Concluding Remarks

This article proposes a method for adjusting the functional dimensions of a chair to any human body size range based only on human height. This is an example of the use of biomimetics, consisting of the use of calculated proportions of the human body to parameterize the dimensions of the chair using seven equations. Table 2 summarizes the proposed formulas to calculate the fundamental chair dimensions.

**Figure 12:**
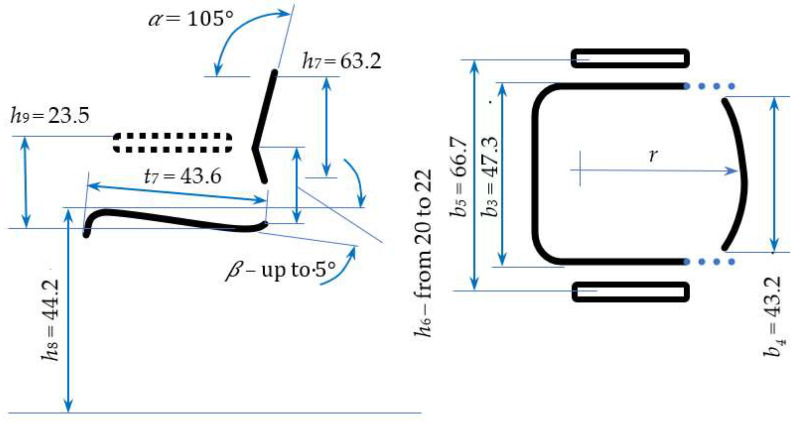
The functional dimensions of a chair for 95% of the adult population (in centimeters).

Figure 12 shows the results of the chair dimension calculations for a wide range of users ranging from 5th-percentile women to 95th-percentile men (source of anthropometric data: [6]). This range covers 95% of the adult population (assuming that the numbers of men and women are equal).

The proposal of chair dimensions presented in Figure 12 covers a wide range of adult users, from a 5th-percentile woman with a height of 150 cm to a 95th-percentile man with a height of 185 cm. Such extreme body dimensions correspond to the fourth size of school chairs (5th-percentile woman) and the seventh size (95th-percentile man) according to EN 1729-1:2015, compared in Table 3.

As can be seen from Table 2, most of the proposed dimensions are within the range given by the standard for school furniture according to EN 1729-1:2015. Two proposed dimensions are increased: *b*_3_ and *b*_5_.

Some limitations should be considered:

- The database used to calculate the body proportions limits the calculation results presented in this article to people with standard body proportions characteristic of adults.
- These limitations can be effectively eliminated by using a more specialized database; for example, the anthropometric database of obese people whose body proportions differ. It is then possible to modify the proportionality coefficients given in Equations (1)–(7) and, as a result, obtain formulas for designing chairs for obese people.

## Figures and Tables

**Table 1:** The links between the measurements of a man and functional dimensions of a chair.

Measurements of a Human	Measurements of a Chair (According to Figure 1)	Comments
*A*—Buttock-popliteal length	*t*_7_, *t*_4_ (indirectly)	*t*_7_ should be less than *A*
*B*—Popliteal height	*h*_8_	*h_8_* should be equal to *B*
*C*—Bi-trochanteric breath or trochanteric width (hip breath)	*b*_3_	*b*_3_ should be greater than *C*
*D*—Elbow-to-elbow breath	*b*_5_	*b*_5_ should be equal to *D*
*E*—Elbow rest height	*h*_9_	*h*_9_ should be equal to *E*
*F*—Sitting height, normal	*h*_7_	*h*_7_ should be less than *F*
*G*—Shoulder breath (bi-acromial breadth)	*b*_4_	*b*_4_ should be equal to *G* or wider
*L.H.*—Lumbar height	*h*_6_	*h*_6_ should be equal to *H*

**Table 2:** The links between measurements of a man and measurements of a chair and proposed formulas.

Reference Measurement of a Human	Example Literature Data	Chair Measurement	Formula	Reference
*A*—buttock-popliteal length	[20,34]	*t*_7_—seat depth	$t_{7}=0.29\cdot H_{5\mathrm{th}\;\mathrm{woman}}$	Height of the smallest expected user, e.g., 5th-percentile woman
*B*—popliteal height	[20,34]	*h*_8_—seat height	$h_{8}=0.25\cdot H_{\mathrm{average}}+2\;\mathrm{cm}$	Height of the average user, with average popliteal height
*C*—bi-trochanteric breadth or trochanteric width (hip breath)	[35,36,37]	*b*_3_—seat width	$b_{3}=0.25\cdot H_{95\mathrm{th}\;\mathrm{woman}}+2\cdot 2\;\mathrm{cm}$	Height of the user with the broadest bi-trochanteric breadth and spacing
*D*—elbow-to-elbow breath	[20,34]	*b*_5_—armrest spacing	$b_{5}=0.27\cdot H_{95\mathrm{th}\;\mathrm{man}}+2\cdot 3\;\mathrm{cm}$	Height of the user with the broadest elbow-to-elbow breath and spacing
*E*—elbow rest height	[20,34]	*h*_9_—armrest height	$h_{9}=0.14\cdot H_{\mathrm{average}}$	Height of the average user, with average elbow rest height
*F*—sitting height	[20]	*h*_7_—backrest height	$h_{7}=0.50\cdot 0.75\cdot H_{\mathrm{average}}$	Height of the average user, with average sitting height
*G*—shoulder breath (bi-acromial breadth)	[6]	*b*_4_—backrest breath	$b_{4}=0.23\cdot H_{95\mathrm{th}\;\mathrm{man}}$	Height of the biggest user, with the broadest shoulder breath
*L.H.*—lumbar height	[17,38]	*h*_6_—lumbar support height	20–22 cm	EN 1729-1

**Table 3:** Comparison of the proposed chair functional dimensions for the 5th–95th-percentile range of the adult population and functional dimensions according to the standard EN 1729-1:2015 [17] (in centimeters).

Chair Measurement	User Height 150–185 (Proposed in Figure 12)	User Height 133–159 (EN 1729-1:2015)	User Height 174–207 (EN 1729-1:2015)
*t*_7_—seat depth	43.6	31	43
*h*_8_—seat height	44.2	38	51
*b*_3_—seat width	47.3	34	40
*b*_5_—armrest spacing	66.7	39–44	51–57
*h*_9_—armrest height	23.5	19	25
*h*_7_—backrest height	63.2	min. 10	min. 10
*b*_4_—backrest breath	43.2	27	36
*h*_6_—lumbar support height	20–22	19	22

## Data Availability

Not applicable.
